# Vasculopathy, Immunodeficiency, and Bone Marrow Failure: The Intriguing Syndrome Caused by Deficiency of Adenosine Deaminase 2

**DOI:** 10.3389/fped.2018.00282

**Published:** 2018-10-18

**Authors:** Pui Y. Lee

**Affiliations:** Division of Allergy, Immunology and Rheumatology, Boston Children's Hospital, Boston, MA, United States

**Keywords:** DADA2, vasculitis, vasculopathy, immunodeficiency, bone marrow failure, cytopenias

## Abstract

Deficiency of adenosine deaminase 2 (DADA2) is a monogenic form of systemic vasculopathy that often presents during early childhood. Linked to biallelic mutations in *ADA2* (previously *CECR1*), DADA2 was initially described as a syndrome of recurrent fever, livedo racemosa, early-onset strokes, and peripheral vasculopathy that resembles polyarteritis nodosum. However, the wide spectrum of clinical findings and heterogeneity of disease, even among family members with identical mutations, is increasingly recognized. Evidence of systemic inflammation and vasculopathy is not uniformly present in DADA2 patients and some can remain asymptomatic through adulthood. Humoral immunodeficiency characterized by low immunoglobulin levels and increased risk of infection is another common feature of DADA2. Variable cytopenias including pure red cell aplasia that mimics Diamond-Blackfan anemia can also be primary manifestations of DADA2. How defects in a single gene translate into these heterogeneous presentations remains to be answered. In this review, we will summarize lessons learned from the pleiotropic clinical manifestations of DADA2.

## Introduction

Vasculitis is group of disorders characterized by inflammation of blood vessels leading to organ dysfunction. Given the ubiquitous presence of blood vessels, vasculitis can result in a wide array of clinical manifestations from the pathologic consequences of inflammation, hemorrhage, and/or ischemia (1). Vasculitides are traditionally classified based on the size of affected vessels, involvement of specific organs, findings on microscopic examination, and presence of other associated etiologies (2, 3).

Most adult vasculitides can be seen in the pediatric setting; the annual incidence of childhood vasculitis is estimated to be 20 per 100,000 individuals under 17 years of age (4). Despite the advances in the diagnosis and treatment, the etiology of most vasculitides remains to be determined. These conditions share features of autoimmunity caused by aberrant activation of adaptive immunity, as illustrated by IgA vasculitis and anti-neutrophil cytoplasmic antigen (ANCA)-associated vasculitis. Similar to other autoimmune diseases, associations with human leukocyte antigen (HLA) variants have been reported in small, medium, and large vessel vasculitis (5). On the other hand, vessel wall inflammation also exhibits features of autoinflammation triggered by components of the innate immune system such as neutrophils and complement (6). Supporting this view, manifestations of vasculitis have been reported in a number of monogenic autoinflammatory syndromes (7, 8).

Deficiency of adenosine deaminase 2 (DADA2) is a unique monogenic autoinflammatory disease that often presents as childhood-onset small and medium vessel vasculitis. Initially described in 2014, DADA2 is now recognized as a mimic of polyarteritis nodosum (PAN) with primary features of early-onset strokes, peripheral vasculopathy and systemic inflammation (9, 10). Subsequent studies in the field have greatly expanded the spectrum of pathology caused by DADA2 (11). Beyond its early description as a monogenic vasculitis, DADA2 is also associated with immunodeficiency and hematologic defects. The heterogeneity of DADA2 manifestations is striking as some individuals with biallelic pathogenic mutations experience rapid deterioration and fatal disease during childhood while other can remain asymptomatic through adulthood (12, 13). To date, more than 160 cases of DADA2 have been described in the literature. In this article, we will discuss the biology of ADA2 and review the major clinical manifestations of DADA2.

## Current biology of ADA2

### Gene and expression

Adenosine is a purine nucleoside generated from the hydrolysis of adenine triphosphate (ATP) and degradation of endogenous nucleic acids. Signaling through several receptors, extracellular adenosine has numerous biological roles including regulation of immune functions (14). Adenosine deaminases (ADA) are enzymes that irreversibly convert adenosine to inosine and 2′-deoxyadenosine to deoxyinosine as a key step in purine metabolism. ADA1, a 40 kD monomeric protein encoded by *ADA* on chromosome 20, is an intracellular enzyme present in most cell types. The absence of ADA1 results in cytotoxic levels of 2′-deoxyadenosine and deoxyATP, leading to severe combined immunodeficiency (SCID) due to death of developing lymphocytes (15).

ADA2 was discovered later on the basis of residual enzymatic activity in patients with ADA1 deficiency (16). The gene that encodes ADA2 in humans was not identified until two decades later by a group of geneticists studying a development disorder called cat eye syndrome. This condition is associated with duplication of a 1.1 megabase region on chromosome 22q11.2, termed cat eye syndrome critical region (CECR) (17). McDermid and colleagues identified a number of candidate genes within this cluster, including a homolog of adenosine deaminase-related growth factor (ADGF) described in insects, which they named *CECR1* (18).

In 2005, Zavialov and Engstrom purified the protein responsible for ADA2 activity in the plasma (19). Mass spectrometry of this protein matched the product of *CECR1* and showed extensive peptide homology to the chicken homolog of ADA2. Unlike the ubiquitous expression of ADA1, ADA2 is expressed primarily by monocytes and macrophages (20, 21). Recently, the HUGO Gene nomenclature committee (HGNC) officially changed the gene symbol to *ADA2*, replacing the former nomenclature of *CECR1, ADGF*, and *IDGFL* (insect-derived growth factor like) used in earlier studies (Table 1).

**Table 1:** Comparison of human adenosine deaminase enzymes.

**Gene symbol**	**ADA1**	**ADA2**	**ADAR**
HGNC ID	189	1839	225
Previous nomenclature	–	CECR1, IDGFL, ADGF	IFI4, G1P1
Chromosome location	20q13.11	22q11.1	1q21.3
Molecular weight	~40 kD	~57 kD	~136 kD
Active form	Monomer	Homodimer	Homodimer
Optimal pH	7.0–7.5	6.6	Unknown
Location	Cytoplasmic	Extracellular	Nuclear
Primary substrate	Adenosine, 2′-deoxyadenosine	Adenosine	Adenosine on RNA
Km (adenosine)	20–50 μM	2.2 mM	Not known
RNA binding domain	No	No	Yes
Cellular expression	Ubiquitous	Monocytes, macrophages, lower in lymphocytes	Ubiquitous
Specific inhibitor	EHNA	Not known	Not known
Glycosylation	No	Yes	Not known
Cellular receptor	CD26/DPPIV	Not known	Not known
Clinical phenotype of deficiency in humans	SCID, neurologic impairment	Vasculopathy, bone marrow failure, immunodeficiency	Aicardi-Goutières syndrome

*HGNC, HUGO Gene Nomenclature Committee; EHNA, erythro-9-(2-hydroxy-3-nonyl) adenine; CECR1, cat-eye syndrome critical region candidate 1; IDGFL, insect-derived growth factor-like; ADGF, adenosine deaminase-related growth factor; DPPIV, Dipeptidyl Peptidase IV; SCID, severe combined immunodeficiency*.

### Protein structure

The structure of ADA2 was solved by Zavialov et al. (22). Consistent with its function as an extracellular protein, ADA2 possesses an N-terminal signal peptide for entry into the endoplasmic reticulum. The protein has a dimerization domain, a putative receptor binding (PRB) domain and a catalytic domain that shares about 20% amino acid identity with the same domain of ADA1. The formation of homodimers with a molecular weight of ~110 kD is required for the enzymatic function of ADA2 *in vitro* (22). Unlike ADA1, ADA2 is heavily glycosylated and N-linked glycosylation is necessary for proper trafficking to the extracellular space (23). ADA2 has also been shown to bind to the surface of various immune cells, possibly through the PRB domain (24). The structural differences between ADA1 and ADA2 are highlighted in Table 1.

### Functional considerations

ADA2 possesses much lower affinity for adenosine compared to ADA1 (16), which raises the question of whether it truly has ADA activity at physiologic concentrations of adenosine (25). Notably, ADA2 is insensitive to the ADA1 inhibitor EHNA, a property that allows discrimination of ADA isoenzyme activity in biological samples. Although both enzymes are conserved through evolution, differences in their cell type of origin, cellular localization, and substrate-binding affinity suggest that ADA1 and ADA2 are not functionally redundant.

Elevated plasma ADA2 concentrations are associated with infection, autoimmune diseases, and malignancy, but whether ADA2 plays a role in these inflammatory processes is not clear (20, 26, 27). *In vitro* studies with human cells have suggested a potential roles of ADA2 in macrophage differentiation and angiogenesis (28, 29). ADA2 orthologs in insects also have growth factor activity that promotes cell proliferation dependent on the ability to metabolize adenosine (30). The precise physiologic function(s) of ADA2 in humans remains a subject of active investigation.

### Quantification of ADA2 activity

Plasma ADA2 activity can be quantified by determining the release of ammonium during the adenosine to inosine conversion (31). Direct measurement of reaction product inosine (in combination with the downstream product hypoxanthine) is also possible using high performance liquid chromatography (9). In both methods, the specific activity of ADA2 is distinguished from ADA1 activity using the ADA1 inhibitor EHNA. While it is possible to quantify ADA2 protein by enzyme-linked immunosorbent assay or western blotting, these methods are not commonly used clinically.

### Other ADA enzymes

It is worth noting that human cells express additional ADA enzymes with nucleic acid binding properties (32). ADAR (ADA acting on RNA), the best-studied member of this family, resides in the nucleus and catalyzes the conversion of adenosine to inosine on RNA (Table 1). Among its many biological roles, ADAR edits endogenous double-stranded RNA to prevent activation of the innate immune system (33). Deficiency of ADAR causes Aicardi-Goutières syndrome characterized by childhood-onset encephalopathy and overproduction of type-I interferons (34). Despite the similarities in enzymatic function, ADAR bears minimal structural resemblance to ADA1 or ADA2.

## The discovery of DADA2

The clinical relevance of human *ADA2* was not appreciated until the discovery of individuals with deleterious mutations in this gene. In 2014, two groups independently described a novel syndrome of systemic inflammation and vasculitis caused by mutations in *ADA2* (9, 10). Zhou and colleagues studied several young children with recurrent fevers, early-onset strokes, livedo racemosa, hepatosplenomegaly, and systemic vasculopathy. Using whole exome sequencing (WES), they determined biallelic loss-of-function *ADA2* mutations in 9 patients and named the condition DADA2. The authors also described the first experimental model of the disease using zebrafish, which showed intracranial hemorrhage and neutropenia after knockdown of the ADA2 homolog (9).

In parallel, Navon Elkan and colleagues studied cases of a medium-vessel vasculitis that closely resembles polyarteritis nodosum (PAN) in the Georgian Jewish population as well as additional patients from Germany and Turkey (10). These patients had recurrent fever, livedo, skin ulceration, neuropathy and aneurysms, among the most prevalent findings. WES identified loss-of-function mutations in *ADA2* and biallelic mutations were identified in 21 patients, with the majority under 10 years of age. The mortality of this condition was highlighted by two deaths in early childhood.

These two landmark studies established DADA2 as a monogenic cause of childhood-onset vasculitis. Over the past few years, our understanding of the clinical spectrum of DADA2 has advanced significantly, with more than 30 publications describing over 160 patients with confirmed mutations world-wide. The collective experience from these studies has been instrumental in guiding treatment approaches. Although the pathogenesis of DADA2 is still under investigation, anti-TNF (tumor necrosis factor) agents seem to be most effective to manage the inflammatory vasculopathy, while hematopoietic stem cell transplantation (HSCT) provides an option for definitive cure (35). Therapeutic options for DADA2 were recently reviewed (11) and will not be extensively discussed here.

## DADA2 as a monogenic cause of vasculopathy

**Figure 1:**
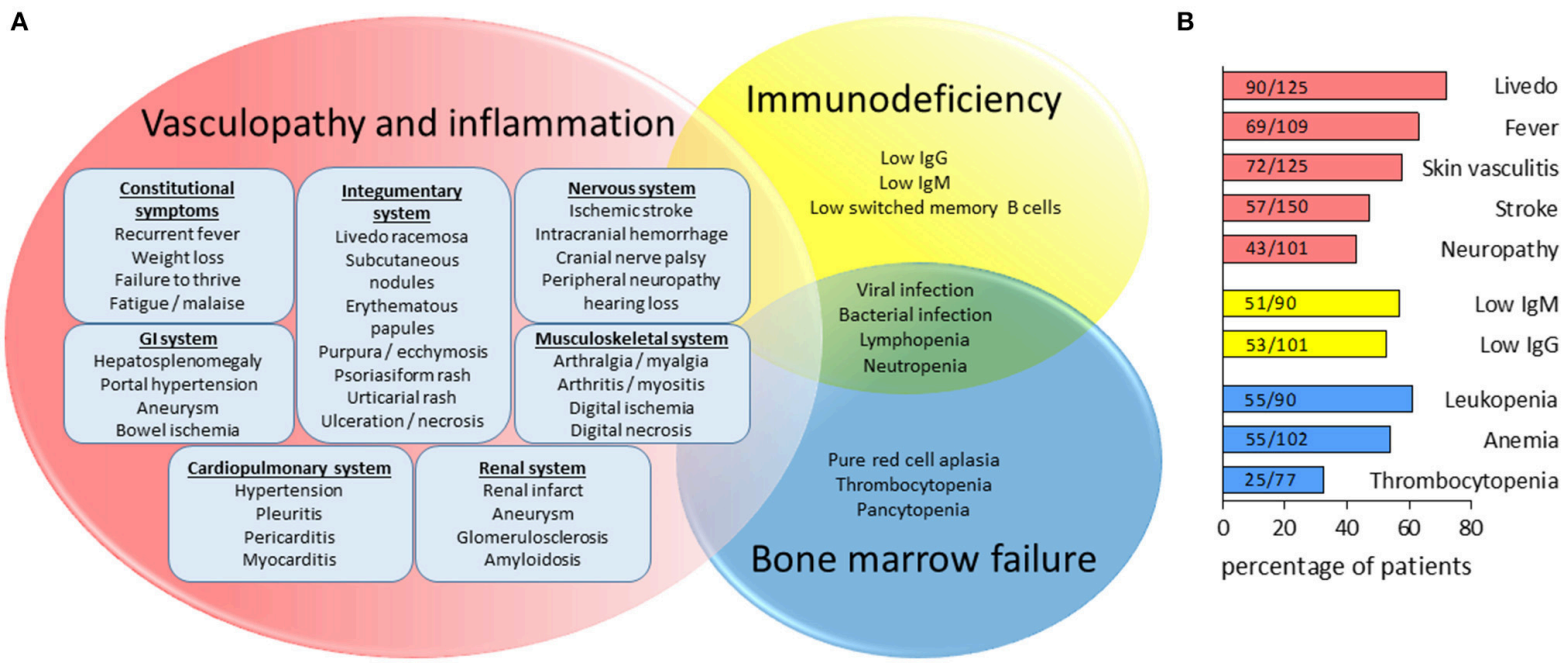
Pleiotropic manifestations of DADA2. **(A)** Depiction of overlapping features of inflammatory vasculopathy, immunodeficiency and hematologic defects in DADA2. **(B)** Bar graph illustrating the estimated percentage of patients with the common clinical features seen in DADA2. As displayed by the Fractions in the graph, estimates are based on the number of patients reported to have the specific clinical features in the literature (160 cases published prior to July 1, 2018) divided by the total number of patients included in the studies in which the parameters were assessed. The category “stroke” include both ischemic strokes and hemorrhagic strokes.

The clinical manifestations of DADA2 are summarized in Figure 1. Consistent with the initial studies, vascular involvement is the hallmark of DADA2 and features of vasculitis or vasculopathy have been described in more than 75% of cases. Organs affected include skin, brain, GI tract, and kidneys. The distribution of medium and small vessel involvement mimics the pattern seen in PAN (10). Not surprisingly, DADA2 patients are often diagnosed with PAN before the diagnosis is confirmed by gene sequencing or plasma activity assay. Caorsi and colleagues reviewed 48 cases of childhood PAN in Italy and found 15 patients with biallelic ADA2 mutations (36). Furthermore, DADA2 has also been found in cases previously diagnosed as Sneddon syndrome, an adult-onset vasculopathy characterized by livedo and strokes (37).

### Skin manifestations

When compared to other patients with PAN, DADA2 patients had earlier disease onset and greater prevalence of skin and neurologic manifestations (36). Overall, skin involvement is the most common manifestation of DADA2, reported in nearly 90% of cases. Based on studies with adequate description of skin findings, livedo racemosa and livedo reticularis are the most common observations (72%), followed descriptions of cutaneous vasculitis (57%) including subcutaneous nodules, petechiae/purpura, ulcerations, and necrosis. Non-specific erythematous maculopapular rash, urticaria, and psoriatic lesions are less common. Biopsy of inflammatory skin lesions typically demonstrates evidence of vasculitis, with variable descriptions including non-granulomatous necrotizing arteritis, leukocytoclastic vasculitis, and panniculitis (9, 10, 38).

### Neurologic manifestations

Neurologic complications of DADA2 include ischemic strokes, intracranial hemorrhage, and a wide range of neuropathy. Ischemic strokes have been reported in more than 50 patients (~38%) and many have experienced recurrent episodes. In some cases, stroke is the initial feature that leads to the diagnosis (12, 39). The severity of strokes varies from small lacunar infarcts with minimal sequelae to larger infarcts leading to permanent neurologic impairments and even death (40). Transient ischemic attacks without radiographic findings have also been reported (41). As brain imaging is not routinely performed in the absence of neurologic findings, subclinical infarcts may escape detection and the prevalence of strokes in this disease may be underestimated.

Intracranial hemorrhage or hemorrhagic strokes have been reported in at least 20 cases. The severity again ranges from subclinical to life-threatening hemorrhage that requires urgent evacuation. Structural abnormalities, such as aneurysms of the cerebral vasculature and basal ganglia calcification seen in interferonopathies are typically absent (42). Whether intracranial hemorrhage is a spontaneous phenomenon in DADA2 or secondary to ischemic stroke conversion remains a question. While coagulopathy is uncommon in this disease, complicating this determination is the use of anticoagulants and/or anti-platelet agents in some patients after an ischemic stroke. Interestingly, radiographic evidence did not suggest cerebral vasculitis in 5 patients with intracranial hemorrhage, and biopsy from 2 of these patients revealed extravasation of red blood cells from small vessels without findings of inflammation (9). In the absence of inflammation, vasculopathy rather than vasculitis may be a more accurate description of the pathology in some cases. A disconnect between brain hemorrhage and inflammatory features of DADA2 is also evident in a case where recurrent spontaneous hemorrhage occurred after effective management of systemic inflammation (23).

### Neuropathy

Diverse manifestations of central and peripheral neuropathy have been reported in patients with DADA2. In fact, neuropathy is another distinguishing feature of DADA2 compared to other cases of childhood PAN, but its connection with vasculopathy is not entirely clear (36). Cranial nerve (CN) palsies can involve CN III, IV, VI, and VII, and several patients experienced neurosensory hearing loss. Ophthalmologic complications including vision loss, central retinal artery occlusion, optic nerve atrophy, uveitis, diplopia, nystagmus, and strabismus have all been described (9, 40). Peripheral neuropathy is noted in half of patients in some cohorts and spastic paraplegia has been reported as a presenting symptom of DADA2 (36, 43).

### Other features of inflammation

Consistent with a systemic inflammatory process, most patients experience recurrent fever. Myalgia and arthralgia are also frequent complaints, but arthritis and myositis are less common. Inflammatory markers such as erythrocyte sedimentation rate and C-reactive protein are often elevated in parallel to the clinical features of inflammation. Early studies established hepatosplenomegaly as a common finding of DADA2 and portal hypertension has been noted in several patients (9).

In keeping with the resemblance to PAN, DADA2 can affect small to medium-size vessels in different organs. Inflammation of arteries supplying the extremities results in digital infarct and necrosis. Vasculitis and aneurysm of the gastrointestinal vasculature leads to bowel perforation and intestinal hemorrhage. Renal aneurysm and infarction have been reported in a handful of cases (10). Hypertension can be seen independent of other renal pathology and may be a risk factor for the development of stroke and intracranial hemorrhage (13, 36, 51). Lastly, rare inflammatory manifestations including pleuritis, pericarditis, myocarditis, meningitis, and amyloidosis have been reported in patients with DADA2 (9, 10).

## Immunodeficiency associated with DADA2

In the initial description of DADA2, Zhou et al. noted mild immunodeficiency with low IgM levels as a clinical feature in 5 patients (9). Schepp and colleagues subsequently found compound heterozygous *ADA2* mutations with absent plasma ADA2 activity in an adult with hypogammaglobinemia and recurrent respiratory infection (44). Expanding on this work, they screened 181 patients with antibody deficiencies and found 9 additional patients with DADA2. Recurrent infection, rather than the classic features of inflammation and vasculopathy, was the primary presentation for more than half of this cohort (45). These patients generally present later in life and several have no clinical evidence of vasculitis. Importantly, low IgG and IgM levels are common features of DADA2, with or without concurrent findings of vasculopathy (Figure 1). Across studies that quantified immunoglobulins, low IgG and/or IgM levels are reported in ~67% of DADA2 patients. The prevalence of IgG deficiency, IgM deficiency, and combined antibody deficiency are ~52, 57, and 35%, respectively.

Both B cell intrinsic and extrinsic factors may contribute to the low immunoglobulin levels. Switched memory B cells are markedly decreased in DADA2 patients and *in vitro* stimulation demonstrates reduced antibody production (9, 45). Defective class-switching, however, cannot account for deficient IgM production. Reduced number of antibody producing cells may be a contributing factor, as DADA2 is associated with lymphopenia (further discussed in the next section). Schepp et al. demonstrated an inverse correlation between CRP and immunoglobulin levels in one patient, suggesting that the inflammatory milieu of DADA2 may directly compromise the B cell compartment (45). This intriguing observation contradicts the observation in pediatric autoimmune conditions, where hypergammaglobulinemia is recognized as a feature of active disease (46).

On the other hand, immunologic studies in DADA2 have not revealed significant impairments in the T cell compartment. Naïve and memory T cell activation, mitogen-induced proliferation, and *ex-vivo* cytokine production by CD4^+^ and CD8^+^ T cells were comparable in patients with DADA2 and control subjects (9). Since specific defects in T cell function are not apparent, infection related to suboptimal T cell function in patients with DADA2 may be better explained by a quantitative defect due to generalized lymphopenia.

Viral and bacterial respiratory tract infections and herpes infection are common in DADA2 patients with antibody deficiencies (45). Infection of the brain and meninges, gastrointestinal tract, urinary tract have also been reported. The risk of infection is escalated further by immunosuppressive agents used to treat the inflammatory features of the disease, although the risk may be partially mitigated if the humoral defects are driven by inflammation. Fungal and mycobacterial infections are unusual in DADA2 but have been noted in cases with severe neutropenia or pancytopenia (47, 48). Fungal infection was also reported in two sibling with a large genomic deletion that spans *ADA2* and the adjacent gene *IL-17RA*, in line with the role of IL-17 in fungal immunity (49).

## Hematologic manifestations of DADA2

The spectrum of hematologic involvement adds another intriguing dimension to our understanding of DADA2 (Figure 1). Severe reduction in erythrocytes, leukocytes, and platelets have all been reported and hematologic defects can be the presenting finding, even without features of inflammation or vasculopathy. Reduced number of immune cells further fuels the infection risk posed by low immunoglobulin levels. Notably, it is often the severity of anemia or pancytopenia that drive the necessity of curative treatment with HSCT (35).

Anemia is present in >50% of cases, often with a severity extending beyond what is expected for anemia of chronic inflammation. Several patients with DADA2 presented with pure red cell aplasia with transfusion dependence that mimics Diamond-Blackfan anemia (47, 50). Genetic evaluation, typically by WES show biallelic *ADA2* mutations while DBA associated mutations in ribosomal proteins and *GATA1* are absent.

Leukopenia occurs in about 60% of cases and variably affect myeloid and lymphoid cells. Thrombocytopenia is less common, described in ~32% of cases. These hematologic defects often do not occur alone as multiple cases of pancytopenia have been reported. Bone marrow biopsy typically demonstrates hypoplasia of the affected lineage(s), suggesting a defect in cell production. Lymphoid cell aggregates in the bone marrow have been documented, although their significance is unclear (48, 50). Evidence of cell consumption is generally absent, but two cases of macrophage activation syndrome has been described (9, 51).

How a plasma protein produced mainly by monocytes and macrophages affects multi-lineage hematopoiesis is unclear. Knockdown of ADA2 in the Zebrafish model was shown to cause neutropenia, supporting an intrinsic role of ADA2 in normal hematopoiesis (9). Whether human ADA2 functions as a cellular growth factor similar to the insect orthologs remains to be seen. *In vitro*, human ADA2 can promote T cell-dependent monocyte maturation (28), though a broader mechanism is necessary given the multi-lineage involvement.

## Rare presentations of DADA2

The clinical spectrum of DADA2 spans beyond the three major phenotypes discussed thus far. Blurring the line the between autoinflammation and autoimmunity, several patients display prominent features of systemic lupus erythematous including the presence of specific autoantibodies and a type I interferon signature (52, 53). Such presentation bears some resemblance to Aicardi-Goutières syndrome associated with mutations in *ADAR* (43). More recently, cases of lymphoproliferative disease with resemblance to large granular lymphocyte leukemia have been described in a Finnish cohort of DADA2 patients (54).

## Genotype to phenotype correlations

Missense mutations, frameshift mutations, splicing defects and deletions have been described in pathogenic *ADA2* mutations. Mutations in all structural domains of ADA2 have also been reported. Neither the type of mutation or location of the mutation seems to correlate with the diverse manifestations of DADA2. Homozygosity of most common pathogenic variants G47R or R169Q explains more than 60 cases of DADA2, but the clinical findings are highly variable among family members that share the same homozygous mutations (10, 12, 51). The amounts of residual ADA2 activity, rather than genotype, may be more predictive of phenotype (12).

It is also important to recognize that individuals (including adults) with confirmed biallelic mutations and absent plasma ADA2 activity can remain entirely asymptomatic (13). These individuals are often found on the basis of having affected family members. On the other hand, some features of the disease including lacunar strokes has been described in heterozygous individuals (9, 55). Additional studies are needed to determine the clinical significance of ADA2 haploinsufficiency.

## Conclusion

Advances in genomic analysis have significantly improved our ability to diagnose monogenic disorders with variable presentations. Less than 5 years after the discovery of DADA2, the clinical spectrum of the disease has drastically expanded from simply a monogenic form of childhood-onset PAN. The pleiotropic presentations of the disease span many sub-specialties, including rheumatology, immunology, neurology, dermatology and hematology in pediatrics as well as in adult medicine. Given the multi-organ involvement of DADA2, recognizing the diverse manifestations is a crucial step toward timely diagnosis and management of this potentially fatal but often treatable syndrome.

## Author contributions

The author confirms being the sole contributor of this work and has approved it for publication.

### Conflict of interest statement

The author declares that the research was conducted in the absence of any commercial or financial relationships that could be construed as a potential conflict of interest.
